# Secondary Metabolomic Analysis and In Vitro Bioactivity Evaluation of Stems Provide a Comprehensive Comparison between *Dendrobium chrysotoxum* and *Dendrobium thyrsiflorum*

**DOI:** 10.3390/molecules28166039

**Published:** 2023-08-13

**Authors:** Lihang Xie, Jinyong Huang, Tingjian Xiong, Yao Ma

**Affiliations:** 1 Academy of Medical Sciences, Zhengzhou University, Zhengzhou 450000, China; xielihang@zzu.edu.cn (L.X.); xiaoraoxiong55@163.com (T.X.); 2 School of Life Sciences, Zhengzhou University, Zhengzhou 450001, China; jinyhuang@zzu.edu.cn; 3 Henan Funiu Mountain Biological and Ecological Environment Observatory, Nanyang 473000, China; 4 School of Agricultural Sciences, Zhengzhou University, Zhengzhou 450001, China

**Keywords:** *Dendrobium chrysotoxum*, *Dendrobium thyrsiflorum*, secondary metabolomic, antioxidant, enzyme inhibition, cytotoxicity

## Abstract

The stems of *Dendrobium chrysotoxum* (DC) are commonly used as health-promoting foods due to their excellent biological activities. However, the stems of *D. thyrsiflorum* (DT) are often used to meet the scarcity of DC in production because of their highly similar morphology. However, the related metabolomic and bioactive information on the stems of DC and DT are largely deficient. Here, secondary metabolites of DC and DT stems were identified using an ultra-performance liquid chromatography-electrospray ionization-mass spectrometry, and their health-promoting functions were evaluated using several in vitro arrays. A total of 490 metabolites were identified in two stems, and 274 were significantly different. We screened out 10 key metabolites to discriminate the two species, and 36 metabolites were determined as health-promoting constituents. In summary, DT stems with higher extract yield, higher total phenolics and flavonoids, and stronger in vitro antioxidant activities demonstrated considerable potential in food and health fields.

## 1. Introduction

The stems of most *Dendrobium* species native to China have been used as health-promoting foods due to the presences of abundant active constituents (i.e., anthocyanins, and polysaccharides) in the materials [1,2,3]. Among these health-promoting *Dendrobium* species, only five species have been recorded in the Chinese Pharmacopoeia (Chinese Pharmacopoeia Commission, 2020). Within this, *Dendrobium chrysotoxum* (DC) is a widely known species for anti-inflammatory, antitumor, hypoglycemic, and antioxidant functions of the stems [4,5]. However, the DC stems are in short supply due to the scarcity of resources, and *D. thyrsiflorum* (DT) stems are consciously or unconsciously regarded as alternatives due to the high similarity in morphologies of species recorded in the Pharmacopoeia [6]. At present, the DT species is known as an endemic herb and important garden flower distributed in southeast China [7]. The phytochemical and bioactive information of DT stems is inadequate, hindering the development of the materials.

Phytochemical and pharmacological investigations of DC and DT stems have disclosed some differences in active constituents and health-promoting functions, but the studies focus on only a single species, explaining inaccurate differences between DC and DT stems due to the influences of sampling, treatment, test method, and even cultivated environment in different studies [6,8]. Moreover, variations in the health-promoting functions of stems between two species remain unclear since only a handful of studies have fully investigated the constituents and bioactivities of DT stems. Thus, a comprehensive comparison, including metabolites and bioactivities in the stems, of two species is urgently needed, subsequently resulting in great benefits for the further development of two plants.

Secondary metabolites play essential roles in the health-promoting functions of plant materials [9,10]. Widely targeted metabolomic technology, covering a wide range of metabolites, has been successfully applied to detect metabolites in various plants [11,12]. The profiling of metabolites coupled with chemometrics could successfully highlight the key phytochemical differences in investigated materials. Moreover, several in vitro tests have been developed to assess the bioactivity of natural extracts [2,13,14]. Thus, reliable metabolomic technology and proven in vitro assay methods could feasibly be used for the comprehensive comparison of metabolites and bioactivities between DC and DT stems.

To fully understand the differentiation between DC and DT species, ultra-performance liquid chromatography-electrospray ionization-tandem mass spectrometry (UPLC-ESI-MS/MS) was conducted to qualitatively and quantitatively analyze the metabolites in the stems of the two species. Several in vitro antioxidant activities combined with enzymes and cell-inhibitory properties were used to evaluate the health-promoting functions of the stems. Moreover, the health-promoting constituents were explored to reveal the reasons for the differences in health-promoting functions between DC and DT stems. Our results provide a comprehensive comparison that highlights metabolite and health-promoting differences between DC and DT stems. In particular, the study develops some biomarkers for the differentiation of DC and DT stems, and further provides valuable information and critical guidance for both species in the development of functional foods.

## 2. Results

### 2.1. Yield, Total Phenolic, and Flavonoid Content of Stem Extracts

**Figure 1:**
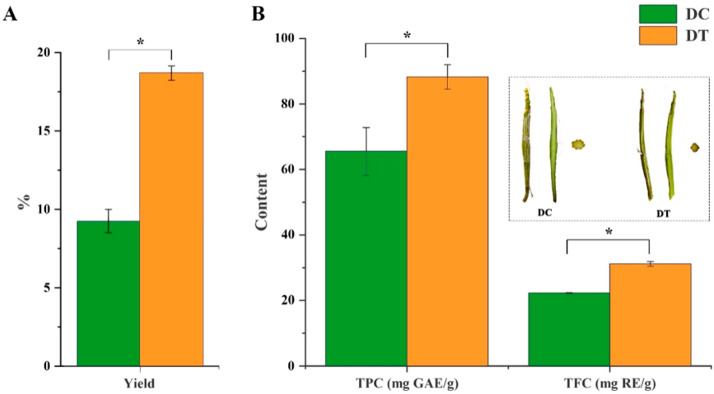
The yields, total phenolic, and flavonoid content in the extracts: (**A**) the yields of extracts; (**B**) total phenolic and flavonoid content in the extracts. DC, *D. chrysotoxum*; DT, *D. thyrsiflorum*; mature stems of DC and DT species (longitudinal section and transverse section) in the dotted line; TPC, total phenolic content; TFC, total flavonoid content; GAE, gallic acid equivalent; RE, rutin equivalent; asterisk (*) above the column diagram indicates significant difference between DC and DT species (*p* < 0.01) for the indicator.

A significantly higher yield was found in DT stem extracts (18.8%), which was more than twice the yield of DC stem extracts (Figure 1A). Both TPC (88.23 mg GAE/g DW) and TFC (31.18 mg RE/g DW) in DT stem extracts were significantly higher than those in DT stem extracts (Figure 1B).

### 2.2. Secondary Metabolite Profiling of Stem Extracts

#### 2.2.1. Overall Secondary Metabolite Profiling

**Figure 2:**
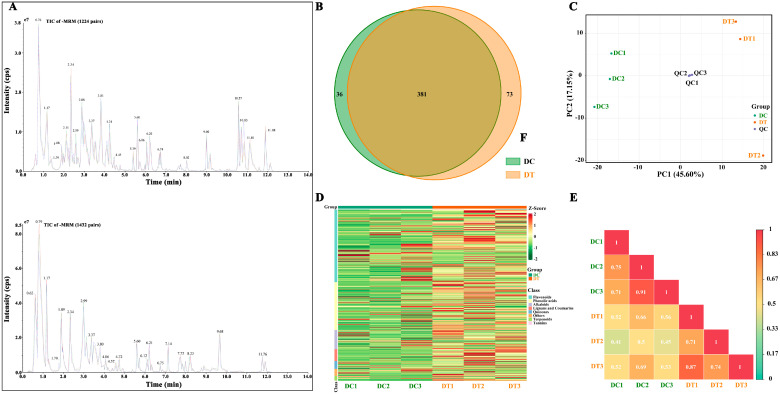
Secondary metabolite profiling of stem extracts: (**A**) total ion chromatogram of different quality control samples using multiple reaction monitoring; (**B**) venn diagram; (**C**) principal component analysis; (**D**) hierarchical cluster analysis; (**E**) correlation analysis. DC, *D. chrysotoxum*; DT, *D. thyrsiflorum*; QC, quality control sample; TIC, total ions chromatogram; +/− MRM, positive and negative multiple reaction monitoring; PC, principal component; the colors in (**D**) represents the level of accumulation of each metabolite, from low (green) to high (red).

To understand the variation of metabolites between DC and DT stems, the secondary metabolites were extracted and extensively analyzed by UPLC-ESI-MS/MS. A total of 490 metabolites were identified in the DC and DT stems (208 flavonoids, 137 phenolic acids, 55 alkaloids, and others). In addition, 381 metabolites existed in both stems, of which 36 and 73 were unique to DC and DT stems, respectively (Figure 2B). Among these unique metabolites, the number of metabolites belonging to flavonoids and phenolic acids was the highest. Erianin presented a high content in DC stems, while no erianin was detected in DT stems. In contrast, 5′-glucosyloxyjasmanic acid and isoquercitrin showed high content in the DT stems, while they were not detected in the DC stems.

To preliminarily comprehend the general differences between the two species and the degree of variation among the samples within the species, PCA was conducted based on all detected metabolites, revealing that the overall metabolic variables contributed to an accumulated variance of 62.8% in the dataset (Figure 2C). Three repetitions in each species were gathered, revealing good repeatability for the samples from each species. Meanwhile, DT and DC samples were clearly separated, revealing significant differences in overall metabolites between DC and DT stems. To eliminate the quantity of pattern recognition, the metabolite data were z-score normalized and then analyzed by HCA (Figure 2D). Two distinct groups associated with DC and DT were revealed, and similar metabolites were found in the stems collected from the same species. Moreover, correlation analysis between samples demonstrated significant differences between DC and DT (Figure 2E).

#### 2.2.2. Determination and Metabolic Pathways of Differential Metabolites

**Figure 3:**
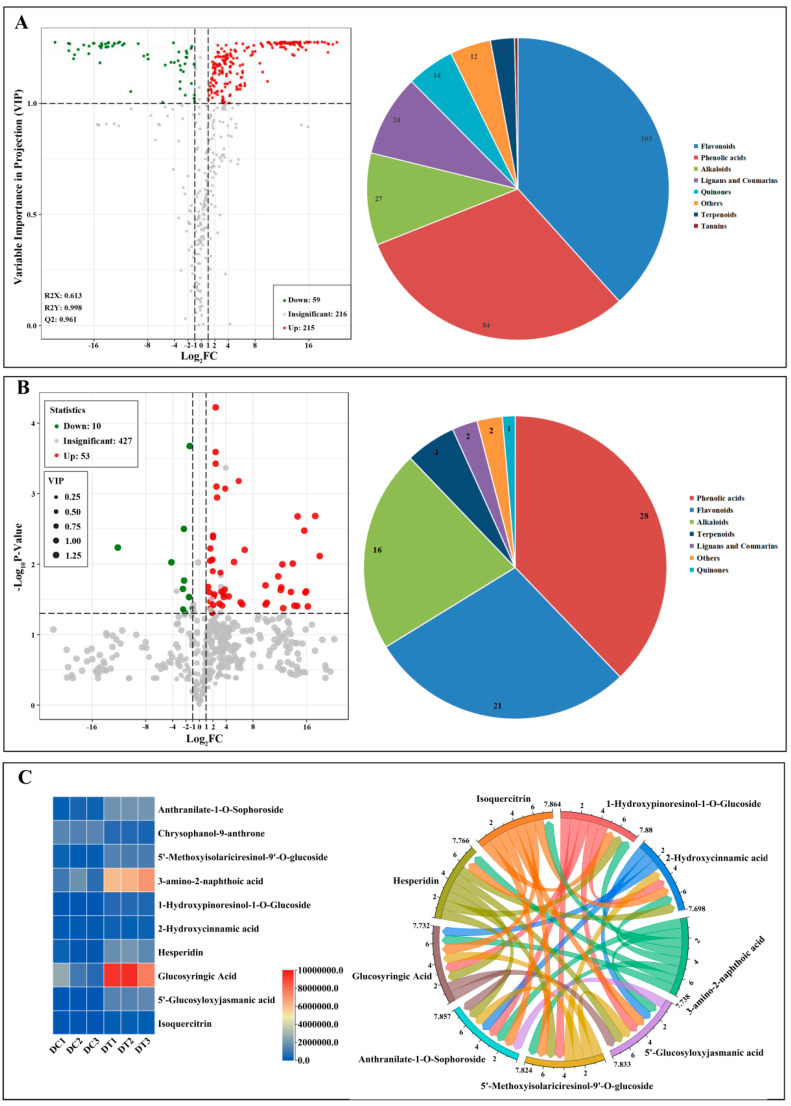
Differential metabolites between *D. chrysotoxum* and *D. thyrsiflorum*: (**A**) volcano plot with fold change; (**B**) volcano plot with fold change and *p* value; (**C**) key differential metabolites with fold change, *p* value, and the false discovery rate.

First, 274 differential metabolites (59 downregulated metabolites and 215 upregulated metabolites) between DC and DT stems were screened out (Figure 3A). More than 20 different classifications can be used to categorize the 274 metabolites: phenolic acids (84) and flavonols (105) made up most of them. Subsequently, 10 downregulated and 53 upregulated metabolites were further screened in consideration of the *p* value (*p* < 0.05) using a *t* test (Figure 3B). In addition, 63 differential metabolites can be categorized into 7 different classes, the majority of which were phenolic acids (28) and flavonols (21). Finally, 10 key differential metabolites (1 downregulated and 9 upregulated) were determined in consideration of a false discovery rate of less than 0.1, and these key differential metabolites demonstrated a strong relationship in the content (Figure 3C).

The pie chart depicts the biochemical categories of the differential metabolites identified between *D. chrysotoxum* and *D. thyrsiflorum*; Figure 3C depicts the content and relationship of key differential metabolites.

**Figure 4:**
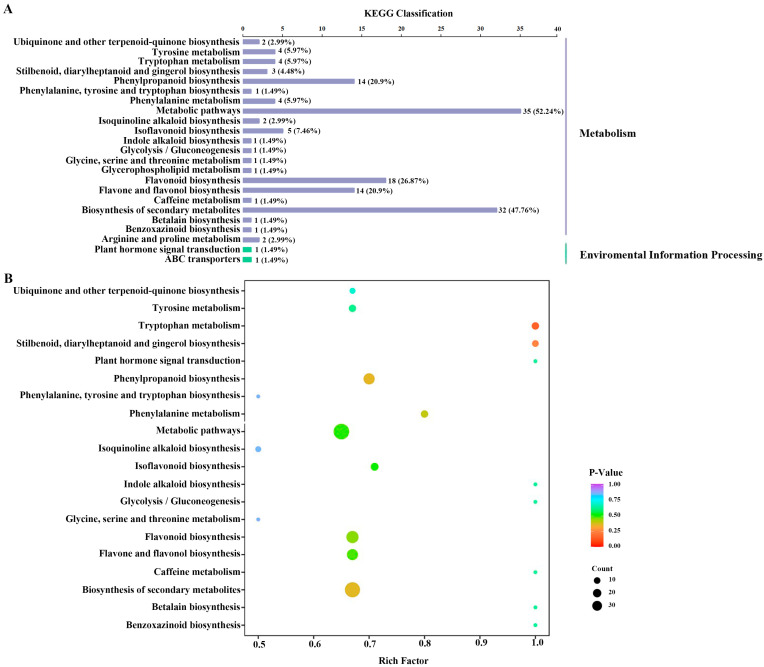
The distribution (**A**) and enrichment (**B**) of metabolic pathways of the differential metabolites. KEGG, Kyoto encyclopedia of genes and genomes.

The majority of the differential metabolites were mapped to ‘metabolism’, especially metabolic pathways and the biosynthesis of secondary metabolites, while a few of the metabolites were mapped to environmental information processing (Figure 4A). Furthermore, the variations in metabolic pathways between DC and DT stems were determined by KEGG pathway enrichment analysis (Figure 4B).

#### 2.2.3. Determination of the Key Health-Promoting Constituents

Most *Dendrobium* species stems are currently regarded as outstanding functional foods for human health mostly because of their high concentrations of bioactive constituents. However, the key health-promoting constituents in the DC and DT stems have not been identified. Therefore, the TCMSP database was used to search for the active constituents using these annotated metabolites in the DC and DT stems. The results showed that 35 of the 490 annotated metabolites were identified as the active constituents of TCMs (Table S1). In addition, erianin, which is a well-known compound in *Dendrobium* stems, was also designated as an active constituent, although it was not found in the chemical compositions of TCMs. Finally, 36 metabolites were identified as the key health-promoting constituents in *Dendrobium* stems, of which 6 constituents (catechin, chlorogenic acid methyl ester, erianin, kaempferide, kaempferol-3-o-neohesperidoside, and neochlorogenic acid) and 7 constituents (apigenin, 6-demethoxycapillarisin, isorhamnetin, kaempferol-7-o-rhamnoside, rhamnetin, syringin, and tamarixetin) specifically existed in DC and DT stems, respectively. The 36 metabolites consisted of 21 flavonoids, 5 phenolic acids, 3 terpenoids, 3 alkaloids, 2 lignans and coumarins, 1 quinone, and 1 other compound, suggesting that many other nonflavonoids in DC and DT stems also exhibited human health-promoting functions, mainly phenolic acids, terpenoids, and alkaloids. These key active constituents were related primarily to therapies for cancer, cardiovascular diseases, Alzheimer’s disease, diabetes, and inflammation, indicating that the metabolites in DC and DT stems were the main health-promoting compounds for human health.

### 2.3. In Vitro Bioactivity of the Stem Extracts

#### 2.3.1. In Vitro Antioxidant Activity

**Figure 5:**
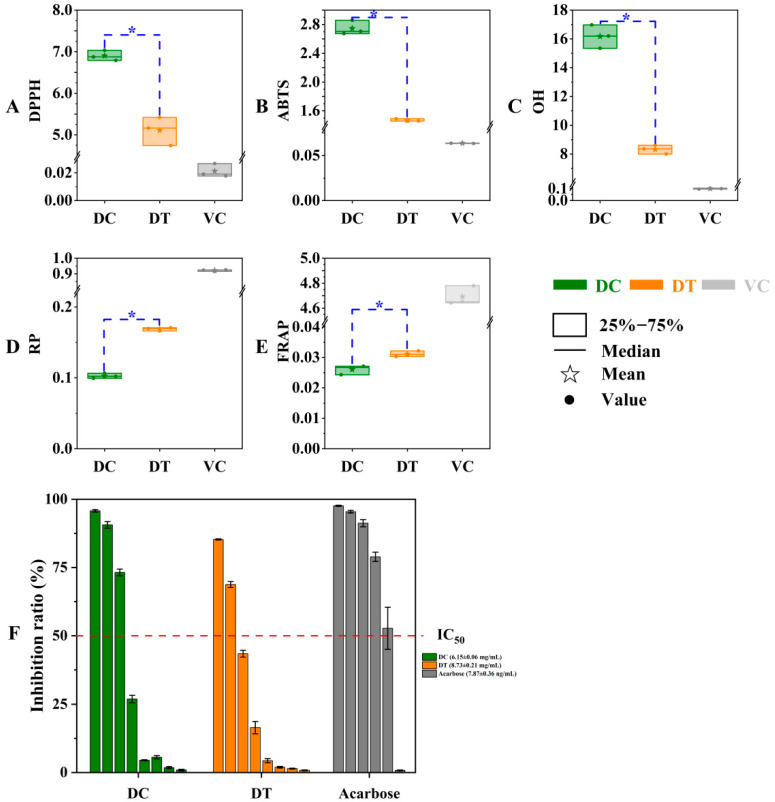
In vitro antioxidant and α-glucosidase inhibition activity of stem extracts of *D. chrysotoxum* and *D. thyrsiflorum*. (**A**) DPPH radical scavenging activity; (**B**) ABTS radical scavenging activity; (**C**) OH radical scavenging activity; (**D**) reducing power; (**E**) ferrous reducing antioxidant power; (**F**) α-glucosidase inhibition activity. DPPH, 2,2-diphenylpicrylhydrazyl; DPPH, 2,2-diphenyl-1-picrylhydrazyl; ABTS, diammonium 2,2′-azino-bis (3-ethylbenzothiazoline-6-sulfonate); OH, hydroxyl; RP, reducing power; FRAP, ferrous reducing antioxidant power; DC, *D. chrysotoxum*; DT, *D. thyrsiflorum*; asterisk (*) above the column diagram indicates significant difference between DC and DT species (*p* < 0.01) for the indicator; IC50, the half maximal inhibitory concentration.

In our work, the antioxidant differences between DT and DC stems were estimated based on DPPH, ABTS, and OH scavenging activities together with reductive power and ferric ion-reducing antioxidant power (Figure 5). As expected, the radical scavenging activities and reducing capacity of stem extracts were markedly lower than those of ascorbic acid. The IC50 values of solvents extracted from stems differed significantly between DC and DT species, and the extracts of DT stems showed better scavenging capabilities of DPPH, ABTS, and OH radicals than the extracts of DC stems. The values of extracts from stems differed significantly between DC and DT species, and the extracts of DT stems showed better antioxidant capacity with regard to the result of power-reducing and ferric-ion-reducing antioxidant ability.

#### 2.3.2. α-Glucosidase Inhibition Assay

In the obtained extracts, the α-glucosidase inhibitory rate decreased gradually in a concentration-dependent manner. The α-glucosidase inhibitory activity of DC stems with a lower IC50 (6.15 ± 0.06 mg/mL) was better than the α-glucosidase inhibitory activity of DT stems (IC50, 8.73 ± 0.21 mg/mL), but the inhibitory difference between the two species was not significant (Figure 5F).

#### 2.3.3. Cytotoxicity of Stem Extracts

**Figure 6:**
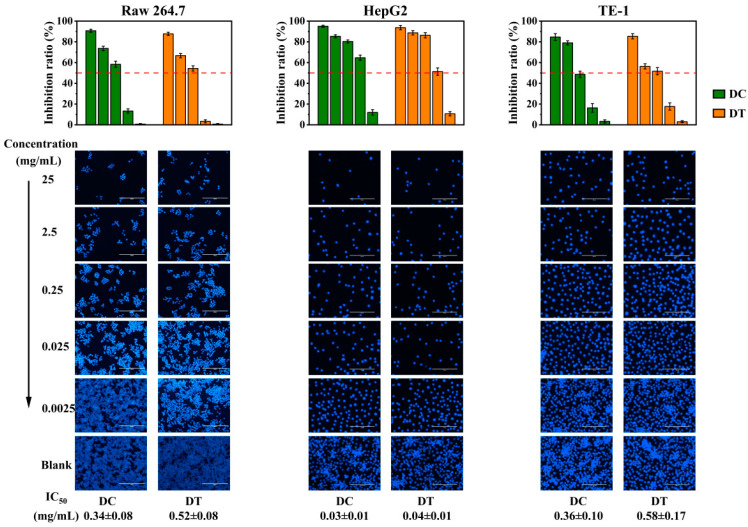
Cytotoxicity of *D. chrysotoxum* and *D. thyrsiflorum* stem extracts. DC, *D. chrysotoxum*; DT, *D. thyrsiflorum*; IC50, the half maximal inhibitory concentration; bar: 100 µm.

The in vitro cytotoxicity of the extracts from DC and DT stems against RAW264.7, HepG2, and TE-1 cells through MTT assay is shown in Figure 6. The inhibitory effects of DC and DT stems against RAW264.7, HepG2, and TE-1 cells were dose dependent, and the differences in IC50 values against the three cell lines between DC and DT stems were not significant. The IC50 values of DC and DT stems against RAW264.7 cells were 0.34 ± 0.08 and 0.52 ± 0.08 mg/mL, respectively. And the IC50 values of DC and DT stems against TE-1 cells were 0.36 ± 0.10 and 0.58 ± 0.17 mg/mL, respectively. In contrast, the IC50 values of DC and DT stems against HepG2 cells were 0.03 ± 0.01 and 0.04 ± 0.01 mg/mL, respectively, which were lower than the corresponding values of RAW264.7 and TE-1 cells. The extracts of DC and DT stems showed stronger cytotoxicity against HepG2 cells than against RAW264.7 and TE-1 cells.

## 3. Discussion

The yield of extracts for plant materials is a crucial aspect when they are applied in product processing [15]. Thus, DT stems with higher yields in extracts demonstrated a larger potential as raw material to extract active constituents. The key active constituents that exist in plant extracts, which are mainly classified as flavonoids and phenolics, have demonstrated various types of biological activities [16]. In this study, The TPC and TFC differences in extracts may be caused by many factors, including plant species, sample treatment, and testing methods [17]. The selected samples in this study were manufactured by the same producer at the same time and treated with the same method to eliminate the effects of storage time, sample treatment and testing methods, and planting environment on metabolites. The results demonstrated the yield, TPC, and TFC differences in stem extracts caused by *Dendrobium* species. Extracts of DT stems, which demonstrated a higher yield and more TPC and TFC, exhibited great potential for food and health fields. 

The number of metabolites identified was significantly higher than that previously recognized in *Dendrobium* species [18], indicating that widely targeted metabolomic technology was a reliable method for thoroughly identifying the metabolites in these plants. Moreover, erianin has been extracted from different *Dendrobium* species and has been found to possess many biological characteristics for preventing and treating cancers with multiple signaling pathways [19]. 5′-Glucosyloxyjasmanic acid has been proven to possess diverse types of activity, i.e., anticancer, anti-inflammatory, and cosmetic activity [20]. Isoquercitrin, commonly found in medicinal herbs, displays many types of bioactivities both in vitro and in vivo, i.e., against cancer, diabetes, and allergic reactions [21]. Our results demonstrated the variation of erianin, 5′-glucosyloxyjasmanic acid, and isoquercitrin between DC and DT stems. In addition, PCA, HCA, and correlation analysis suggested that these stems of the two species had distinct metabolites, despite both of the species being from the same section of the genus *Dendrobium*. Similar chemometric methods were also used to distinguish *D. huoshanense* and *D. officinale* based on establishing nitrogen-containing compounds [22]. Metabolic profiling based on PCA, HCA, and correlation analysis could be an efficient measure for the differentiation of different *Dendrobium* species.

Differential metabolite analyses showed that chrysophanol-9-anthrone, the only downregulated key differential metabolite, is a very unstable form, and it can easily be converted to chrysophanol, which has obvious hepatotoxicity and nephrotoxicity [23]. Thus, DT stems could be regarded as a prospective substance for food supplementation for safety and beneficial pharmacological activity. In addition, the route that differed most noticeably between DC and DT stems was tryptophan metabolism. Previous studies found that auxins were synthesized mainly through tryptophan-dependent or tryptophan-independent pathways in all plant parts [24], which might explain why the stem length of DT was generally longer than the stem length of DC.

The *Dendrobium* stems are promising natural resources for antioxidants [3], but the antioxidant activities of DT and DC stems are rarely reported, especially for DT stems. Thus, the antioxidant difference between DT and DC stems is still not clear. The total antioxidant capacity of a plant extract cannot be accurately measured by any antioxidant test [25]. Both free radical scavenging activity and reducing power must be taken into consideration in a thorough antioxidant test. The reducing power and ferric-ion-reducing antioxidant power are usually detected to evaluate reducing capacity, and higher values indicate a higher antioxidant capacity [15,26]. The differentiation of antioxidant capacity might be caused by different types and amounts of metabolites in botanical extracts [27,28]. It has been noted in the past that phenolic compounds and antioxidant actions are positively correlated [25,26]. This result confirmed the agreement between the active compounds and antioxidant activity, where the extracts of DT stems showed higher yield, TPC, and TFC. Thus, DT rather than DC stems could be regarded as a potential antioxidant material with regard to the different antioxidant assays.

α-Glucosidase can break down polysaccharides in the gut, increasing the blood sugar level. Inhibiting the enzyme will result in lower sugar absorption during digestion. Acarbose, a common α-glucosidase inhibitor, shows a decrease in postprandial blood glucose, but it results in flatulence and abdominal pain due to the microbiota fermenting undigested carbohydrates in the large intestine [25]. Some traditional medicinal materials have demonstrated considerable α-glucosidase inhibitory activity to control high blood glucose levels [29,30]. In general, polyphenols (such as flavonoids and anthocyanins) were reported to be effective α-glucosidase inhibitors [31,32]. Stilbene constituents in the genus *Dendrobium* have been reported to exhibit strong α-glucosidase effects [33]. A total of 4 stilbene constituents (resveratrol, dihydroresveratrol, 4′-methoxyresveratrol, and 3,4′-dihydroxy-5-methoxystilbene) were detected among DT and DC stems, and similar contents of these stilbene constituents may result in the inapparent difference in α-glucosidase inhibitory activity between DT and DC stems, although DC stems with lower TPC and TFC demonstrated a better α-glucosidase inhibitory activity compared to DT stems. Thus, DT and DC stems are substituted as α-glucosidase inhibitors.

DC and DT stems were identified with an abundance of flavonoids and phenolic acids, which are well-explored molecules with excellent anticancer efficacy [34]. The difference in cytotoxicity between DC and DT stems could be attributed to their difference in these secondary metabolites. Only bibenzyls and bibenzyl derivatives in the genus *Dendrobium* have been reported to exhibit potent cytotoxicity against cancer cell lines [35]. A similar content of bibenzyls and bibenzyl derivatives in stems may result in an inapparent cytotoxicity difference between DT and DC species. In addition, the bioactivity of many molecules is modulated by matrix effects that can also affect their bioavaibility. A suitable matrix could further improve the bioactivity and bioavailability of molecules. In the future, the underutilized DT stem could be considered to solve the problem of insufficient DC resources due to the presence of valuable phytochemical profiles in the case of similar cytotoxicity between DC and DT stem extracts.

## 4. Materials and Methods

### 4.1. Chemicals and Reagents

All mass spectrometric grade reagents (methanol, acetonitrile, and formic acid) were bought from Sigma-Aldrich Trading Co., Ltd. (Shanghai, China), while chromatographic grade reagents (rutin, gallic acid, and acarbose) and analytical grade reagents, including sodium nitrite, aluminum chloride, sodium hydroxide, sodium carbonate, ascorbic acid, Folin–Ciocalteu reagent, 2,2-diphenyl-1-picrylhydrazyl (DPPH), diammonium 2,2′-azino-bis (3-ethylbenzothiazoline-6-sulfonate) (ABTS+•), potassium persulfate, phosphate-buffered saline (PBS), salicylic acid, ferrous sulfate, tripyridine triazine (TPTZ), ferric chloride, sodium acetate, potassium ferricyanide, hydrogen peroxide solution, hydrochloric acid, acetic acid, trichloroacetic acid, α-glucosidase, p-nitrophenyl-β-d-glucopyranoside (pNPG), and 4′,6-diamidino-2-phenylindole (DAPI), were purchased from Yuanye Bio-Technology Co., Ltd. (Shanghai, China). Mouse mononuclear macrophage leukemia cells (RAW 264.7 cells), human hepatoma cells (HepG2 cells), and human esophageal carcinoma cells (TE-1) were provided by Wanwu Bio-Technology Co., Ltd. (Hefei, Anhui, China).

### 4.2. Collection of Plant Material and Sample Preparation

The plants of DC and DT were cultivated under uniform growth condition for five years at Ning’er Hani Yi Autonomous County, Pu’er City, Yunnan, China (101°03′8.79″ N, 23°03′16.52″ E). In 2021, five stems of each species were collected on April 15 when the stems were marketable. The collected stems were cut into pieces and freeze-dried using a YTLG-10A vacuum freeze drier (Shanghai Yeto Technology Co., Ltd., Shanghai, China). An amount of 200 mg of powdered stems were mixed with 5 mL of methanol (60%, *v*/*v*) in a centrifuge tube. Then, the tubes were kept at 30 °C for 30 min in a XM-5200 UVF ultrasound bath (Xiaomei Ultrasonic Instrument Co., Ltd., Suzhou, China) and centrifuged at 12,000 rpm for 10 min at 4 °C (Eppendorf Centrifuge 5424R, Eppendorf China Ltd., Shanghai, China). The supernatant liquid was collected, and the residual was redissolved using 5 mL of 60% methanol. The extraction process was repeated three times, and the mixed supernatant liquids were filtered with quantitative filter papers. The solution was set to a volume of 15 mL; 5 mL of the solution was used for phytochemical analysis, and the other was evaporated using a reduced-pressure rotating evaporator and freeze-dried. The yield of the extract was expressed by the percentage of each extract in dry raw material (%). A total of six extracts were obtained for three repetitions of the two species, and then the extracts were refrigerated at 4 °C in colored vials before use.

### 4.3. Determination of Total Phenolics and Total Flavonoids in the Extracts

The total phenolic content (TPC) and total flavonoid content (TFC) was estimated using the Folin–Ciocalteu assay and aluminum chloride colorimetric method according to the outlined procedures, respectively [17]. The TPC of each sample was calculated from the standard gallic acid curve at 700 nm using a TECAN SPARK multimode reader (TECAN, Männedorf, Switzerland), and it was expressed as gallic acid equivalent (GAE) per gram dry stem weight (mg GAE/g DW). The TFC of each sample was calculated from the standard rutin curve at 510 nm, and it was expressed as rutin equivalent (RE) per gram dry stem weight (mg RE/g DW).

### 4.4. Metabolomic Profiling of the Extracts

#### 4.4.1. The Qualitative and Quantitative Analysis of Metabolites

The extract solution was filtered through a membrane (0.22 μm) before UPLC-ESI-MS/MS analysis (UPLC, SHIMADZU NexeraX2, Shimadzu, Kyoto, Japan; MS, Applied Biosystems 4500 Q TRAP, Applied Biosystems, Waltham, MA, USA) [36]. A quality control sample was prepared from a mixture of six extracts to check the consistency of the samples. One quality control sample was inserted for every 3 test extracts, and three quality control samples were performed. An overlapping total ion chromatogram for quality control samples illustrated a good repeatability and stability of metabolite identification, proving the validity of the test sample data (Figure 2A).

The following conditions were applied to the analysis: mobile phases, solvent A (pure water with 0.1% formic acid) and solvent B (acetonitrile with 0.1% formic acid); column, Agilent SB-C18 (1.8 m, 2.1 mm, 100 mm); gradient program with a flow rate of 0.35 mL/min, 0–11.0 min 95–5% A, 11.0–12.0 min 5–5% A, 12.0–12.1 min 5–95% A, 12.1–15.0 min 95–95% A; temperature, 40 °C; injection volume, 4 μL.

The ESI parameters were as follows: turbospray ion source temperature, 550 °C; ion spray voltage, 5500 V (positive ion mode)/−4500 V (negative ion mode); ion source gas, 50, 60, and 25.0 psi for gas I, gas II, and curtain gas, respectively. Instrument and mass calibration were carried out with 10 and 100 mol/L polypropylene glycol solutions in the triple series quadrupole mass spectrometry (QQQ) and linear ion trap modes, respectively. As part of numerous reaction monitoring investigations, the collision gas (nitrogen) was adjusted to 0.34 bar when the QQQ scans were obtained.

#### 4.4.2. Determination and Annotation of Differential Metabolites

The relative contents of metabolites in DC and DT stems were further compared according to the outlined procedure [14]. First, the differential metabolites were screened out when the fold change of metabolites in DT stems compared to DC stems was ≥2 (upregulated) or ≤0.5 (downregulated) based on log2 transformation before and then mean centering values of metabolites, and then the variable importance in projection (VIP) value of the orthogonal partial least squares discriminant analysis (OPLS-DA) was considered (not less than 1). The detected metabolites were annotated using the Kyoto encyclopedia of genes and genomes (KEGG) database, and then they were mapped to the KEGG pathway database. Finally, the key differential metabolites were determined in consideration of a false discovery rate less than 0.1.

#### 4.4.3. Determination of the Key Health-Promoting Constituents

All detected metabolites in DC and DT stems were queried in the traditional Chinese medicine systems pharmacology (TCMSP) database. The metabolites were considered as key health-promoting constituents in DC and DT stems when the parameters of drug-likeness ≥0.14 and oral bioavailability ≥5% were determined [37]. Information on the disorders associated with the health-promoting constituents was also obtained.

### 4.5. Determination of In Vitro Bioactivities for the Extracts

#### 4.5.1. Determination of In Vitro Antioxidant Assays

The free radical scavenging activities of DPPH, ABTS+• and hydroxyl (•OH), reducing power, and ferrous-reducing antioxidant power (FRAP) of the stem extracts were evaluated according to previously published methods with minor adjustments [15]. For these in vitro antioxidant assays, the same volume of methanol was employed as the blank control group, and ascorbic acid was employed as the positive control.

First, 50 μL of different extract solutions were combined with 200 μL of DPPH (40 mg/L) in a 96-well microplate, and the mixture was then kept for 30 min at room temperature in the dark. Then, the absorbance was measured at 517 nm.

Potassium persulfate solution (2.45 mM) and ABTS ammonium (7 mM) were mixed to create the ABTS+• solution, and then the solution was kept at room temperature overnight in the dark. After that, the solution was diluted 20 times with PBS. Then, 200 μL of ABTS+• solution and 50 μL of different extract solutions were added to a 96-well microplate. The absorbance was recorded after 10 min at 37 °C of incubation in the dark. 

The •OH radical scavenging activity was generated through a Fenton reaction. In brief, 50 μL of different extract solutions, 50 μL of ferrous sulfate (5 mM), 50 μL of hydrogen peroxide (1%), and 50 μL of salicylic acid (5 mM) were successively added into a 96-well microplate. The absorbance was recorded at 510 nm after 15 min of incubation at 37 °C. 

The reducing power was estimated by the Prussian blue method. The extracts (50 μL) were mixed with 200 μL of PBS (0.2 M, pH 6.6) and 200 μL of potassium ferricyanide solution (1%). A volume of 200 μL of trichloroacetic acid (10%) was added to the mixture after it had been kept at 50 °C for 20 min in a water bath. Then, the mixture was centrifuged at 5000 r/min for 10 min, and the supernatant was mixed with 200 μL of distilled water and 50 μL of ferric chloride (0.1%). The absorbance was recorded at 700 nm. 

Acetate buffer (300 mM, pH 3.6), TPTZ reagent (10 mM in 40 mM hydrochloric acid), and ferric chloride (20 mM) were mixed in a ratio of 10:1:1 to create the FRAP reagent, which was then kept at 37 °C for 30 min. Different extracts (50 μL) were mixed with FRAP working solution (200 µL). After incubation at 37 °C for 10 min, the absorbance was measured at 593 nm. The FRAP ability was expressed in mM ferrous sulfate.

#### 4.5.2. α-Glucosidase Inhibition Assay

The α-glucosidase inhibition assay was executed according to the reported method [38]. In brief, 50 µL of α-glucosidase (0.5 U/mL) and 50 µL of different extract solutions were mixed in a 96-well microplate, and the mixture was preincubated at 28 °C for 6 min. Following the preincubation, 50 µL of pNPG (dissolved in PBS) was added to the mixture to start the reaction for 10 min at 37 °C. Then, 100 µL of sodium carbonate (0.2 M) was added to the solution to stop the reaction. The rate of pNPG conversion to p-nitrophenol was calculated by measuring the absorbance of p-nitrophenol at 405 nm. Acarbose was employed as a positive control, and the same volume of PBS was used as the negative control.

#### 4.5.3. Determination of Cytotoxicity

RAW 264.7, HepG2, and TE-1 cells were grown using Dulbecco’s modified Eagle’s medium (plus 10% inactivated fetal bovine serum and 1% penicillin and streptomycin) in an incubator with 5% carbon dioxide at 37 °C, and the solution was replaced once every 2 days. More than 95% of the cells used in the experiment were alive, and all the cells were in the logarithmic growth phase (2.5 × 10^5^ cells/mL).

The DAPI staining method was employed to detect cells [39]. RAW 264.7, HepG2, and TE-1 cells were incubated in a 12-well plate for 24 h, and different extracts (0, 0.0025, 0.025, 0.25, 2.5, and 25 mg/mL) were added. The mixtures were cultured for 24 h. Next, 2.5 μg/mL DAPI (100 μL) was added for 20 min. After the DAPI stain was eliminated, the plate underwent three PBS washes. Finally, the cells were photographed under an AMG Evos M5000 Inverted Fluorescence Microscope (Westover Scientific, Inc., Seattle, WA, USA), and they were counted using ImageJ software (https://imagej.net/ij/index.html, accessed on 10 August 2023).

#### 4.5.4. Calculation of the Half Maximal Inhibitory Concentration

The free radical (DPPH, ABTS+•, and •OH) scavenging rate (%), α-glucosidase inhibition rate, and cytotoxicity were calculated as (Ablank − Asample)/Ablank × 100. The mass concentration of the sample at 50% inhibition is known as the half-maximal inhibitory concentration (IC50), and lower IC50 values indicate a higher inhibitory capacity [3,15]. The inhibitory rates of the samples at eight different concentrations (double half dilution method at 25.00 mg/mL of initial concentration for scavenging rates and α-glucosidase inhibition rate, and decuple dilution method for cytotoxicity) were used to draw the curve. The curves were linearly fitted to obtain the IC50 values of the DPPH, ABTS+•, and •OH free radical arrays, α-glucosidase inhibition rate, and cytotoxicity.

### 4.6. Statistical Analysis

One-way analysis of variance and Pearson’s correlation tests at the 0.01 level were conducted on SPSS 19.0 statistical software (SPSS Inc., Chicago, IL, USA). A column diagram was constructed using Origin 2021 software v2.2.0 (Originlab, Northampton, MA, USA). The filtered metabolite data were submitted to R (www.r-project.org, accessed on 10 August 2023) for principal component analysis (PCA), hierarchical cluster analysis (HCA), and OPLS-DA.

## 5. Conclusions

Systematic and comprehensive studies demonstrated the similarities and dissimilarities of metabolites and in vitro bioactivity between DC and DT stems. In this study, a total of 490 metabolites (208 flavonoids, 137 phenolic acids, 55 alkaloids, and others) were detected in DC and DT stems based on widely targeted metabolomic technology. Among these metabolites, 274 were differential metabolites which were mostly engaged in metabolic pathways and biosynthesis of secondary metabolites. We found 59 metabolites that were significantly lower in the DT stems than that in the DC stems, 10 key differential metabolites were screened out as phytochemical markers for the identification of both species, and 36 metabolites were determined to be key health-promoting constituents. In conclusion, DT stems, possessing higher extract yield, higher TPC and TFC, stronger in vitro antioxidant activities, and similar α-glucosidase and cytotoxicity with DC, exhibited considerable potential for food and health fields. This investigation shed light on the metabolites and bioactivity of DC and DT stems and provided first-hand thorough information on the metabolites with health-promoting properties for humans.

## Data Availability

Data is contained within the article or Supplementary Materials.

## Supplementary Materials

The following supporting information can be downloaded at: https://www.mdpi.com/article/10.3390/molecules28166039/s1, Table S1: Key active constituents in *Dendrobium* stems in the traditional Chinese medicine systems pharmacology database.

## Sample Availability

Samples of the compounds are available from the authors.
