# Supplementary material for: Secondary Metabolomic Analysis and In Vitro Bioactivity Evaluation of Stems Provide a Comprehensive Comparison between Dendrobium chrysotoxum and Dendrobium thyrsiflorum
Source: Molecules. 2023 Aug 13;28(16):6039. doi: 10.3390/molecules28166039 (PMC10458425; doi:10.3390/molecules28166039)
Supplement: Supplementary file 1 [file molecules-28-06039-s001.zip › molecules-2538722-supplementary.pdf]

Table S1 Key active constituents in *Dendrobium* stems in the traditional Chinese medicine systems pharmacology database

Compounds	Class
Betaine	Alkaloids
cis-N-p-Coumaroyltyramine	Alkaloids
p-Coumaroyltyramine	Alkaloids
Chrysin	Flavonoids
Apigenin	Flavonoids
Calycosin	Flavonoids
Kaempferol-7-O-rhamnoside	Flavonoids
Isohyperoside	Flavonoids
Kaempferol-3-O-neohesperidoside	Flavonoids
Hesperidin	Flavonoids
Acacetin	Flavonoids
Catechin	Flavonoids
Diosmetin	Flavonoids
Kaempferide	Flavonoids
Pratensein	Flavonoids
Hispidulin	Flavonoids
Rhamnocitrin	Flavonoids
Quercetin	Flavonoids
6-Hydroxyluteolin	Flavonoids
Morin	Flavonoids
Hesperetin	Flavonoids
Tamarixetin	Flavonoids
Rhamnetin	Flavonoids
Isorhamnetin	Flavonoids
Eucommin A	Lignans and Coumarins
Scopoletin-7-O-glucoside	Lignans and Coumarins
6-Demethoxycapillarisin	Others
Butin	Phenolic acids
Chlorogenic acid methyl ester	Phenolic acids
Syringin	Phenolic acids
Neochlorogenic acid	Phenolic acids
Erianin	Phenolic acids
Emodin	Quinones
Geniposide	Terpenoids
Ursolic acid	Terpenoids
Dehydroabietic acid	Terpenoids
